# Trophoblast Side-Population Markers are Dysregulated in Preeclampsia and Fetal Growth Restriction

**DOI:** 10.1007/s12015-024-10764-w

**Published:** 2024-07-19

**Authors:** Georgia P. Wong, Sunhild Hartmann, David G. Simmons, Sarah Ellis, Olivia Nonn, Ping Cannon, Tuong-Vi Nguyen, Anna Nguyen, Lucy A. Bartho, Stephen Tong, Natalie J. Hannan, Tu’uhevaha J. Kaitu’u-Lino

**Affiliations:** 1 The Department of Obstetrics, Gynaecology and Newborn Health, Mercy Hospital for Women, University of Melbourne, 163 Studley Road, Heidelberg, Victoria 3084 Australia; 2 Mercy Perinatal, Mercy Hospital for Women, Heidelberg, Victoria Australia; 3 Charité – Universitätsmedizin Berlin, corporate member of Freie Universität Berlin and Humboldt-Universität, Berlin, Germany; 4 Experimental and Clinical Research Center, a cooperation between the Max-Delbrück-Center for Molecular Medicine in the Helmholtz Association and the Charité - Universitätsmedizin Berlin, Berlin, Germany; 5 Max-Delbrück-Center for Molecular Medicine in the Helmholtz Association (MDC), Berlin, Germany; 6 DZHK (German Center for Cardiovascular Research), partner site Berlin, Berlin, Germany; 7 School of Biomedical Sciences, University of Queensland, Brisbane, Australia; 8 Olivia Newton-John Cancer Research Institute, Heidelberg, VIC 3084 Australia; 9 School of Cancer Medicine, La Trobe University, Melbourne, VIC 3086 Australia; 10 Division of Cell Biology, Histology and Embryology, Gottfried Schatz Research Center, Medical University of Graz, Graz, Austria

**Keywords:** Trophoblast, Placenta, Preeclampsia, Fetal growth restriction, Pregnancy

## Abstract

Dysregulated progenitor cell populations may contribute to poor placental development and placental insufficiency pathogenesis. Side-population cells possess progenitor properties. Recent human trophoblast side-population isolation identified enrichment of 8 specific genes (*CXCL8, ELL2, GATA6, HK2, HLA-DPB1, INTS6, SERPINE3* and *UPP1*) (Gamage et al. 2020, *Stem Cell Rev Rep*). We characterised these trophoblast side-population markers in human placenta and in placental insufficiency disorders: preeclampsia and fetal growth restriction (FGR). Trophoblast side-population markers localised to mononuclear trophoblasts lining the placental villous basement membrane in preterm control, preeclamptic and FGR placental sections (*n* = 3, panel of 3 markers/serial section). Analysis of single-cell transcriptomics of an organoid human trophoblast stem cell (hTSC) to extravillous trophoblast (EVT) differentiation model (Shannon et al. 2022, *Development*) identified that all side-population genes were enriched in mononuclear trophoblast and trophoblasts committed to differentiation under hTSC culture conditions. *In vitro* validation via 96 h time course hTSC differentiation to EVTs or syncytiotrophoblasts (*n* = 5) demonstrated *ELL2* and *HK2* increased with differentiation (*p* < 0.0024, *p* < 0.0039 respectively). *CXCL8* and *HLA-DPB1* were downregulated (*p* < 0.030, *p* < 0.011 respectively). *GATA6* and *INTS6* increased with EVT differentiation only, and *UPP1* reduced with syncytialisation. *SERPINE3* was undetectable. Trophoblast side-population marker mRNA was measured in human placentas (< 34-weeks’ gestation; *n* = 78 preeclampsia, *n* = 30 FGR, and *n* = 18 gestation-matched controls). *ELL2, HK2* and *CXCL8* were elevated in preeclamptic (*p* = 0.0006, *p* < 0.0001, *p* = 0.0335 respectively) and FGR placentas (*p* = 0.0065, *p* < 0.0001, *p* = 0.0001 respectively) versus controls. Placental *GATA6* was reduced in pregnancies with preeclampsia and FGR (*p* = 0.0014, *p* = 0.0146 respectively). Placental *INTS6* was reduced with FGR only (*p* < 0.0001). This study identified the localisation of a unique trophoblast subset enriched for side-population markers. Aberrant expression of some side-population markers may indicate disruptions to unique trophoblast subtypes in placental insufficiency.

**Graphical Abstract:**

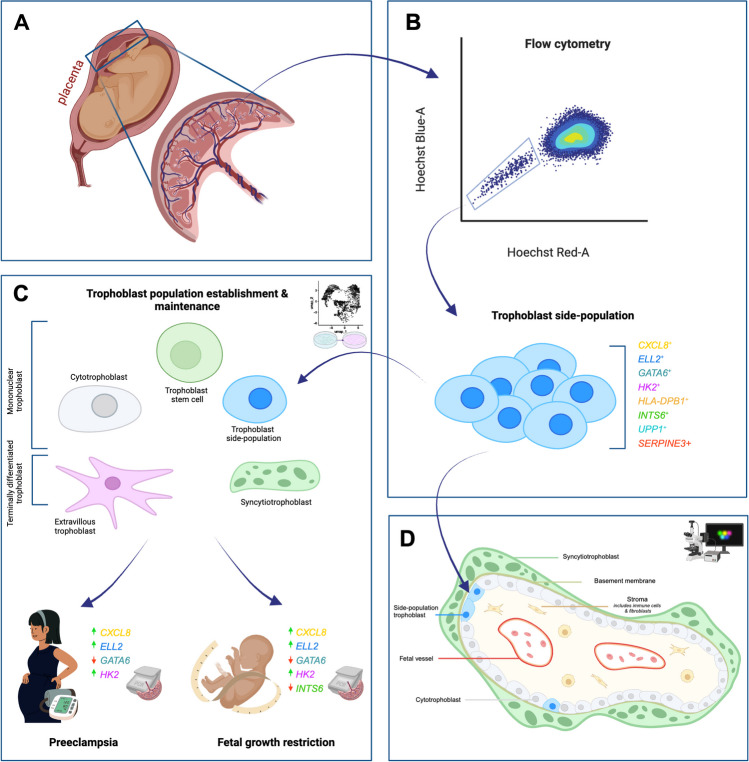

**Supplementary Information:**

The online version contains supplementary material available at 10.1007/s12015-024-10764-w.

## Introduction

The placenta represents the life-support system for a fetus throughout pregnancy. Aberrant placentation may trigger downstream dysfunction, conferring poor nutrient perfusion and vulnerability to the development of placental insufficiency. Preeclampsia and fetal growth restriction (FGR), two prevalent placental insufficiency disorders, can lead towards poor obstetric outcome. Each disease affects between 3–8% of pregnancies worldwide, with an inequitable distribution amongst low-resource settings [1]. In preeclampsia, placental malperfusion incites endothelial dysfunction in the maternal systemic vasculature, manifesting clinically as maternal hypertension and end-organ dysfunction [2]. In FGR, restricted placental function results in the fetus failing to reach its genetically predetermined growth potential *in utero* [3]. FGR is the largest standalone risk factor for stillbirth, with perinatal survival predisposing to lifelong elevated incidences of morbidity and mortality [3]. These two disorders can occur in isolation or in unison, suggesting some elements of molecular mechanisms may be shared. Understanding placental insufficiency pathogeneses is crucial to developing preventative and therapeutic measures that will alleviate the existing and extensive burden of disease.

The placenta facilitates the control of nutrient and waste exchange and is a proxy for developing fetal organs. Therefore, establishment and maintenance of its trophoblast (placental epithelia) sub-populations are critical to a healthy pregnancy. To date, much research effort has focused on understanding the terminal trophoblast phenotypes and their functions. However, more recent attention has been focused on the existence and identification of a distinct pool of placental progenitor cells. Should these progenitor pools become dysregulated, perturbations in differentiation trajectories to mature trophoblast phenotypes may ensue, contributing to placental insufficiency. Identifying and investigating molecular markers that provide indication of these niche cell populations may therefore serve to uncover their roles in placental development and physiology as well as in pathological conditions. These may aid in identifying opportunities to improve detection and intervention for pregnancy complications.

Once classical epithelial progenitor cells commit to a cell fate, they transform into intermediate progenitor cells that undergo rapid proliferation in a process termed ‘transit amplification’ [4, 5]. Historical concepts referred to mononuclear cytotrophoblasts as a bipotential progenitor trophoblast population, however; more recent data suggests that there are likely multiple subpopulations of cytotrophoblasts, with each representing commitment to a more terminally differentiated trophoblast lineage [6, 7]. These differentiated lineages include invasive extravillous trophoblasts (EVTs) and multinucleated syncytiotrophoblasts. Collectively, trophoblasts act to anchor the placenta to the maternal decidua during pregnancy, induce remodelling and by extension enhance capacitance of the maternal blood flow. They also form a specialised transporting barrier, allowing effective maternal-fetal exchange.

Side-population cells are a cell population, that demonstrate progenitor properties of self-renewal and potency, and are enriched for progenitor and stem cell surface markers [8]. They are isolated by excluding the DNA-binding fluorescent Hoechst 33342 dye via expression of ATP-binding cassette cell membrane transporter proteins [9, 10]. Flow cytometry sorts the ‘Hoechst-low’ population, that appears to the ‘side’ of other cell populations, explaining their distinction as ‘side-population’ cells. Recently, the Hoechst 33342 technique yielded ‘side-population trophoblasts’ from the placenta [11]. Side-population trophoblasts persist across all trimesters of pregnancy, with reduced abundance in FGR-affected placentas [12]. This trophoblast population was postulated to include the resident placental progenitor cell population that may be dysregulated in FGR pathogenesis [12]. Isolated side-population trophoblasts are transcriptionally distinct, with increased expression of 8 genes: C-X-C motif chemokine ligand 8 *(CXCL8)*/interleukin-8 *(IL8),* elongation factor for RNA polymerase II 2 *(ELL2),* GATA*-*binding factor 6 *(GATA6),* hexokinase 2 *(HK2),* HLA class II histocompatibility antigen DP β chain 1 *(HLA-DPB1),* integrator complex subunit *6 (INTS6),* serpin family E member 3 *(SERPINE3),* uridine phosphorylase 1 *(UPP1).* However, identification of where these side-population cells reside in placenta remains unknown.

This study aimed to localise the trophoblast side-population cells through expression of their distinct markers in human placenta via multiplexed immunofluorescence (mIF), single-cell transcriptomics, and *in vitro* differentiation studies. Once cell types were discerned, dysregulation of the proposed side-population trophoblast marker panel in human placental lysates were assessed. This sought to shed light on disruption to the establishment and maintenance of placental cell populations that may contribute to placental insufficiency and adverse perinatal outcomes, namely preeclampsia and fetal growth restriction.

## Methods

### Opal™-tyramide Signal Amplification (TSA) Multiplexed Immunofluorescence (mIF) and Image Analysis

The grouping and order of antibodies in each panel were determined with the following considerations: 1) Each antibody required pairing with a fluorophore. Less abundant markers were matched to brighter fluorophores (Opal™ 520 – CXCL8, HLA-DPB1), intermediate abundance (Opal™ 570 – UPP1, INTS6) and more abundant markers were paired with dimmer fluorophores (Opal™ 690 – SERPINE3, HK2). 2) The effects of the number of rounds of heat-induced epitope retrieval (HIER) can degrade or enhance epitope exposure and subsequent staining. This was determined by each antibody stained on serial sections that were subject to either 1 or 3 rounds of HIER. Antibodies that were degraded with multiple HIER treatments resulted in later application and vice versa. 3) To mitigate incomplete stripping between HIER treatments, antibodies localising to the same subcellular compartments (for example, cytoplasm or nucleus), were not applied sequentially [13]. The resulting fluorophore pairings and staining order were as follows: Panel 1 labelled on one serial section) SERPINE3-Opal™ 690, HLA-DPB1-Opal™ 520, and UPP1-Opal™ 570. Panel 2 labelled on a second serial section) HK2-Opal™ 690, CXCL8-Opal™ 520, and INTS6-Opal™ 570. Once optimised, Opal™ multiplexed immunofluorescence (mIF) staining [14] was conducted on formalin-fixed paraffine-embedded (FFPE) sections. Single marker controls were additionally run to ensure consistency between individual and mIF stains. Paraffin embedded placental samples (*n* = 3 biological replicates of placentas obtained from pregnancies that delivered with preeclampsia, FGR or preterm gestation-matched controls) were serial sectioned at 4 µm and collected on SuperFrost™ Plus adhesion microscope slides (ThermoFisher Scientific™). Sections were dried at room temperature overnight. Placental sections were deparaffinised and rehydrated as follows: xylene three times, 100% ethanol twice, 70% ethanol for 3 min each; distilled water 1 min. Side-population markers were localised with the Opal 6-Plex Detection Kit (Akoya Biosciences®, Massachusetts, US) as per manufacturer’s instructions. BLOXALL® Endogenous Blocking Solution and ImmPRESS® HRP Universal Antibody (Vector Laboratories, California, US) were used as an endogenous peroxidase blocking and secondary antibody respectively. The following primary antibodies were utilised each with application at 4 °C overnight: anti-CXCL8 (anti-IL8, Abcam, Cambridge, UK, Cat#Ab106350), anti-HK2 (ThermoFisher Scientific™, Cat#PA5-29326), anti-HLA-DPB1 (Abcam, Cat#Ab157210), anti-INTS6 (Sigma-Aldrich, Missouri, US, Cat#HPA001846), anti-SERPINE3 (Novus Biologicals, Minnesota, US, Cat#NBP2-34209) and anti-UPP1 (Novus Biologicals, Cat#NBP2-30852). Antibodies for ELL2 (Novus Biologicals, Cat#NBP2-55603) and GATA6 (Bio-Techne, Minnesota, US, Cat#AF1700) were unable to be optimised due to limited antibody specificity. Immunofluorescence tyramide signal amplification (TSA) dyes Opal™ 520, Opal™ 570 and Opal™ 690 and counterstaining with Spectral DAPI were applied. Slides were imaged using the Vectra® Polaris™ 3.0 Automated Quantitative Pathology Imaging System, 200 slide (Perkin Elmer, Massachusetts, US). Whole slide analysis was performed for each section at 10 × magnification using the HALO® Image Analysis Platform’s Highplex FL module v4.1.3 (Indica Labs, Albuquerque, US).

### Single-cell RNA Sequencing (scRNA-seq) of an hTSC-derived Organoid Model for Trophoblast Differentiation

To analyse scRNA-seq data, the code previously published (https://github.com/MatthewJShannon) was applied on the dataset (GEO accession number GSE174481) for the pre-processing steps and adjusted for subsequent analysis [15].

Using the Seurat R package (version 5.0.1) [16, 17], 8228 cells were used for pre-processing of the data including doublet removal with DoubletFinder package version 2.0.4 [18]. The remaining 6354 cells were used for downstream analysis. Downstream analysis was performed as previously described [15] including integration of all 6 samples (*n* = 3 biological replicates for each condition), normalisation and single-cell transcriptomics transformation with subsequent clustering using the FindCluster function at a resolution of 1. Dimensionality reduction was performed using the runUMAP function with 33 principal components. Characteristic marker genes for each cell type were used to annotate clusters. Each cluster was annotated with 1 of the following 6 cell identities: mononuclear trophoblast (MNT), characterised by expression of paternally expressed 10 (*PEG10*) and tumour protein 63 (*TP63*); proliferative mononuclear trophoblast (MNTprol) by marker of proliferation Ki-67 (*MKI67*) [19–21], pre-fusion mononuclear trophoblast (MNTpf) by endogenous retrovirus (ERV) group W member 1, ERV group FRG member 1, ERV group V member 1 (*ERVW-1*, *ERVFRD-1*, *ERVV-1*), progenitor EVT (pEVT) by *MKI67*, centromere protein K (*CENPK*), integrin subunit α 2 (*ITGA2*), human leukocyte antigen G (*HLA-G*); invasive EVTs (iEVT) by *ITGA2*, *HLA-G*, matrix metalloproteinase 2 (*MMP2*), v-erb-b2 avian erythroblastic leukemia viral oncogene homolog 2 (*ERBB2*)*,* placenta-specific 8 (*PLAC8*); and syncytiotrophoblasts (STB) by major facilitator superfamily domain-containing protein 2 (*MFSD2A*) and cytochrome P450 aromatase (*CYP19A*) [20, 22–27]. General trophoblast markers: epidermal growth factor receptor, keratin 7, transcription factor AP-2 γ, GATA binding protein 3 (*EGFR*, *KRT7, TFPA2C, GATA3* respectively) were also applied [28, 29]. After cluster annotation, a data subset was created based on EVT-differentiated and undifferentiated samples. Dot plot and feature plots based on the single gene expression and module scores using the AddModuleScore function were generated for the genes *CXCL8 (IL8), ELL2, GATA6, HK2, HLA-DPB1, INTS6, SERPINE3, UPP1* to visualize their respective expression. The full code is available upon request.

### Culture of Human Trophoblast Stem Cells

A human trophoblast stem cell (hTSC, CT30, female) line was imported from the RIKEN BioResource Research Center via the National BioResource Project of MEXT/AMED, Japan (RCB Cat#RCB4938, RRID: CVCL_A7BB) [30]. 24 h after plating, hTSCs were cultured in cell culture media that induced differentiation to either extravillous trophoblasts (EVTs) or syncytiotrophoblasts for 96 h. Cell lysates were collected for RNA extraction and subsequent gene analysis at 0, 48 and 96 h post-introduction to differentiation media. Cell culture experiments were run in technical duplicates and repeated *n* = 5 times (as biological replicates).

### Placental Lysates: Early Onset Preeclampsia & Fetal Growth Restriction

Placental samples were sourced from the Mercy Hospital for Women Tissue Bank (Heidelberg, Victoria, Australia). Samples were donated following informed, written consent. This study was approved by the Mercy Health Human Research Ethics Committee (R11/34).

Placental tissue was obtained from patients who delivered with early-onset preeclampsia (< 34 weeks’ gestation, *n* = 61 biological replicates), FGR (*n* = 12) or both (*n* = 18), compared to placentas collected from gestation-matched control pregnancies (*n* = 18). Control placentas originated from deliveries with normal birth weight centile (> 10th centile for gestational age) that were normotensive and unaffected by chorioamnionitis, as confirmed by placental histopathology. Diagnoses of preeclampsia were made according to the American College of Obstetricians and Gynecologists (ACOG) guidelines (2020) [31]. FGR was defined as < 10th birthweight centile on local birthweight charts [32]. All placentas were obtained as outlined prior [33]. For participant characteristics, refer to Supplementary Table II (Placental RNA < 34 weeks’ gestation).

### RNA Isolation

The GenElute™ mammalian total RNA miniprep kit (Sigma-Aldrich) was used to isolate RNA from placental samples and hTSCs as per manufacturer’s instructions. mRNA concentration quantification was conducted with the Nanodrop ND 1000 spectrophotometer (NanoDrop Technologies Inc), and equivalent amounts converted to cDNA as described below.

### Quantitative Reverse Transcriptase Polymerase Chain Reaction (qRT-PCR)

RNA was reverse transcribed into cDNA with the High-Capacity cDNA Reverse Transcriptase Kit (Applied Biosystems, Massachusetts, US) as per the manufacturer’s instructions. For gene expression, the primers used to target each gene were as follows: *CXCL8* (Assay ID: Hs00174103_m1), *ELL2* (Assay ID: Hs00831747_s1), *GATA6* (Assay ID: Hs00231122)*, HK2* (Assay ID: Hs00606086_m1)*, HLA-DPB1* (Assay ID: Hs03045105_m1)*, INTS6* (Assay ID: Gs00247179_m1)*, SERPINE3* (Assay ID: Hs01391001_m1)*, UPP1* (Assay ID: Hs01066247_m1), TEA domain transcription factor 4 (*TEAD4,* Assay ID: Hs01125032_m1), human leukocyte antigen G *(HLA-G,* Assay ID: Hs03045108_m1), and syndecan-1 *(SDC1,* Assay ID: Hs00896423_m1). Gene expression was quantified through the CFX384 Touch Real Time PCR Detection System (Bio-Rad, California, US) with 10 μL reactions consisting of fluorescein (FAM)-labelled TaqMan Fast Advanced Master Mix (ThermoFisher Scientific™, Massachusetts, US) and each specific primer (Life Technologies, California, US). Quantitative reverse transcriptase polymerase chain reactions (qRT-PCRs) were performed under the conditions as follows: 95° for 20 s, with 40 subsequent amplification cycles to denature for 3 s at 95 °C, with annealing for 30 s at 60 °C. No product was detected in non-template controls. Gene expression was calculated as the geometric mean of cytochrome C1 (*CYC1,* Assay ID: Hs00357717_m1) and DNA topoisomerase I (*TOP1,* Assay ID: Hs00243257_m1) for placental samples, or for *in vitro* studies, glyceraldehyde 3-phosphate dehydrogenase *(GAPDH)* was used for hTSC differentiation to syncytiotrophoblasts, while *CYC1* was utilised for hTSC differentiation to EVTs. Samples were run as technical duplicates, with the average threshold (Ct) value used. Gene expression was normalised to the mean Ct of each control group, with analysis conducted via the 2^−ΔΔCt^ method.

### Statistical Analysis

All *in vitro* experiments were performed with technical duplicates and repeated five times (as biological replicates) unless stated otherwise. Normality and lognormality tests (Anderson-Darling test, D’Agostino and Pearson test, Shapiro-Wilk test, and Kolmogorov-Smirnov test) were conducted to select statically appropriate tests. Data with two unpaired groups were analysed with either an unpaired *t*-test (parametric) or Mann-Whitney test (non-parametric). Analysis of data with more than three groups were analysed with one-way ANOVA (parametric) or Kruskal Wallis tests (non-parametric) with post-hoc analyses to identify differences. Significance value of *p* < 0.05 was used. All statistical analyses were performed on Graph Prism 10.1.0 (GraphPad Software Inc, California, US).

## Results

### Opal™-tyramide Signal Amplification (TSA) Multiplexed Immunofluorescence (mIF) in Placental Sections from Human Preterm Controls, and Pregnancies with Preeclampsia or Fetal Growth Restriction

We first sought to localise the side-population markers to a distinct trophoblast population in human placental samples via mIF. Initially, each antibody was optimised individually prior to the combination staining mIF. Opal staining evaluating co-expression of markers on a specific cell type, such as the side-population, required the selection of fluorophores that had minimal spectral overlap (Opal™ 520, Opal™ 570 and Opal™ 690). Therefore, a maximum of 3 markers plus a DAPI nuclear counterstain could be localised to any one section. Given the limited specificity of the ELL2 and GATA6 antibodies trialled (data not shown), these were not included in the two panels.

**Fig. 1:**
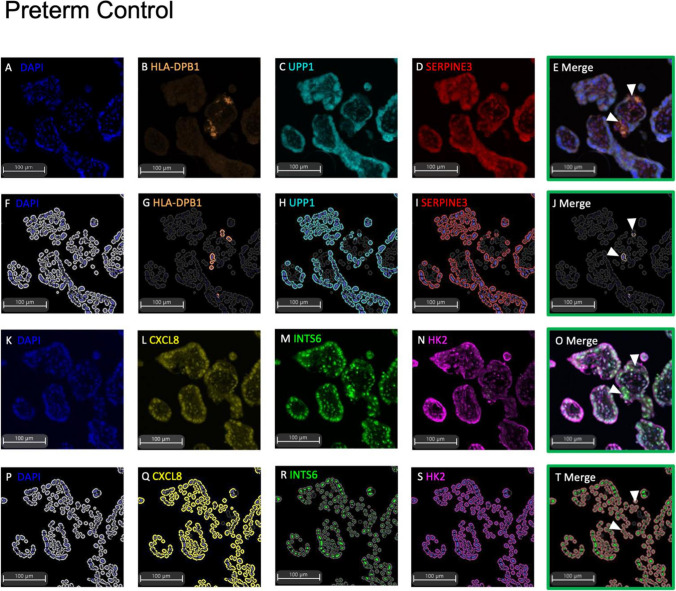
Multiplexed immunofluorescence of trophoblast side-population markers in preterm placental serial sections (< 34-week gestation). One serial section stained for each of Panel 1 and 2. Representative raw images of Panel 1: DAPI nuclear counterstain (**A**), HLA-DPB1 (**B**), UPP1 (**C**), SERPINE3 (**D**), with merge of Panel 1 (**E**). HALO Analysis of positive immunostaining for DAPI **(F)**, HLA-DPB1 (**G**), UPP1 (**H**), SERPINE3 (**I**), and cells expressing co-localisation of Panel 1 markers **(J)**. Representative raw images of Panel 2: DAPI counterstain (**K**), CXCL8 (**L**), INTS6 (**M**), HK2 (**N**), merge of Panel 2 (**O**). HALO analysis of positive immunostaining for DAPI (**P**), CXCL8 (**Q**), INTS6 (**R**), HK2 (**S**), and cells expressing co-localisation of Panel 2 markers (**T**). Representative images of *n* = 3 at 10 × magnification shown

For panel 1 (Fig. 1A-J), in placentas obtained from preterm control, HLA-DPB1 positivity (Fig. 1B & G) was observed in the membrane of stromal immune cells and select trophoblasts lining the borders of villous cross sections. UPP1 (Fig. 1C & H) and SERPINE3 (Fig. 1D & I) localised to the cytoplasm of trophoblasts lining the basement membrane of the placental villi. Similar expression for each marker in panel 1 was observed in each experimental group. In panel 2 (Fig. 1K-T), each marker also demonstrated consistent localisation across all experimental groups. CXCL8 (Fig. 1L & Q) was expressed in the villous cross section in addition to syncytiotrophoblast and select underlying trophoblast layers. INTS6 (Fig. 1M & R) was localised to the nuclei of villous syncytiotrophoblast and underlying cytotrophoblast. INTS6 was also observed in the stroma and endothelial cells lining the placental vasculature (data not shown). HK2 (Fig. 1N & S) appeared to localise to the cytoplasm of similar cell populations to INTS6.

Positivity for both immunofluorescent side-population panels appeared to be specific to select trophoblasts positioned along the basement membrane of the placental villous cross sections. This was observed in the gestation-matched preterm controls. Panel 1 (HLA-DPB1 + /UPP1 + /SERPINE3 +) positivity suggested staining in suspected syncytial knots (Fig. 1E raw, J analysis), but the ability to trace these sections serially with panel 2 (INTS6 + /CXCL8 + /HK2 +) indicated that these were likely false knots that instead reflect syncytial branching points or intervillous bridges (connection points of the syncytioplasms between neighbouring villi) (Fig. 1O raw, T analysis) [34, 35].

**Fig. 2:**
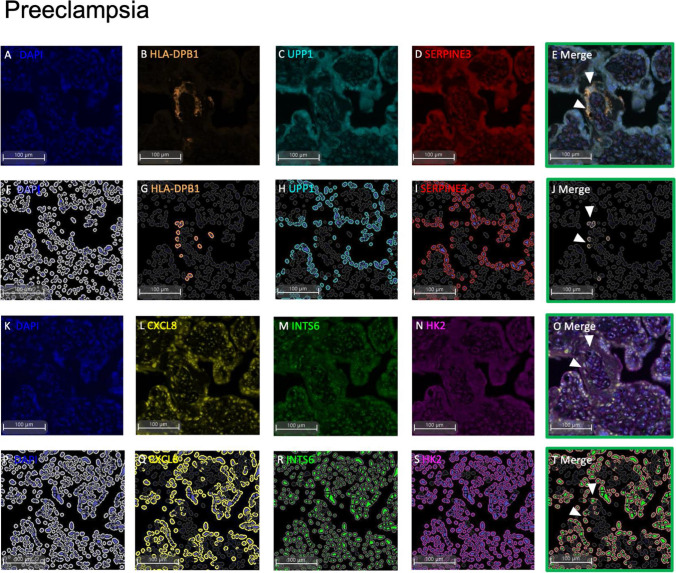
Multiplexed immunohistochemistry of trophoblast side-population markers in placentas from preterm preeclampsia (< 34-week gestation). One serial section stained for each of Panel 1 and 2. Representative raw images of Panel 1: DAPI nuclear counterstain (**A**), HLA-DPB1 (**B**), UPP1 (**C**), SERPINE3 (**D**), with merge of Panel 1 (**E**). HALO Analysis of positive immunostaining for DAPI (**F**), HLA-DPB1 (**G**), UPP1 (**H**), SERPINE3 (**I**), and cells expressing co-localisation of Panel 1 markers (**J**). Representative raw images of Panel 2: DAPI counterstain (**K**), CXCL8 (**L**), INTS6 (**M**), HK2 (**N**), merge of Panel 2 (**O**). HALO analysis of positive immunostaining for DAPI (**P**), CXCL8 (**Q**), INTS6 (**R**), HK2 (**S**), and cells expressing co-localisation of Panel 2 markers (**T**). Representative images of *n* = 3 at 10 × magnification shown

**Fig. 3:**
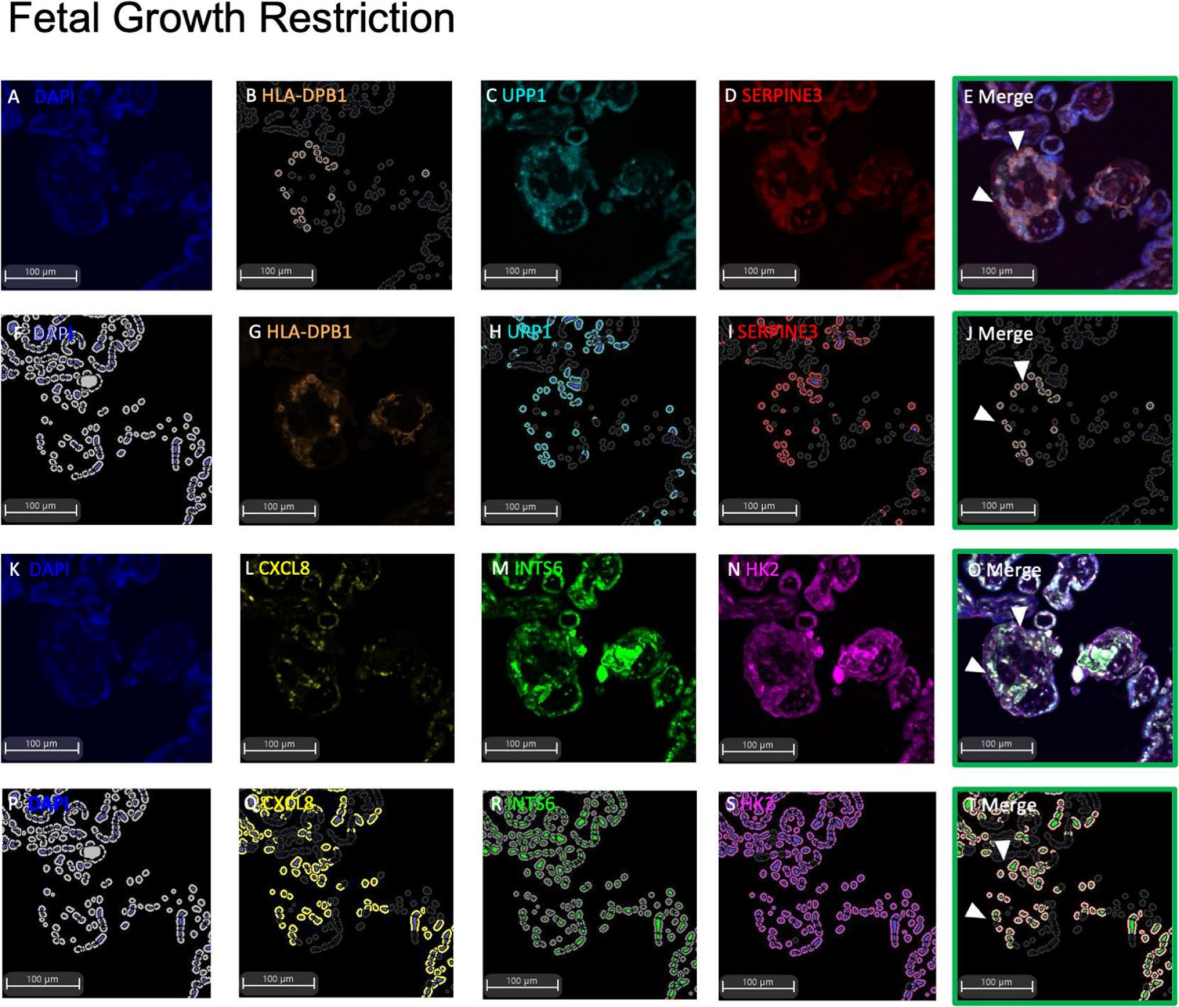
Multiplexed immunohistochemistry of trophoblast side-population markers in placentas from fetal growth restriction (< 34-week gestation). One serial section stained for each of Panel 1 and 2. Representative raw images of Panel 1: DAPI nuclear counterstain (**A**), HLA-DPB1 (**B**), UPP1 (**C**), SERPINE3 (**D**), with merge of Panel 1 (**E**). HALO Analysis of positive immunostaining for DAPI (**F**), HLA-DPB1 (**G**), UPP1 (**H**), SERPINE3 (**I**), and cells expressing co-localisation of Panel 1 markers (**J**). Representative raw images of Panel 2: DAPI counterstain (**K**), CXCL8 (**L**), INTS6 (**M**), HK2 (**N**), merge of Panel 2 (**O**). HALO analysis of positive immunostaining for DAPI (**P**), CXCL8 (**Q**), INTS6 (**R**), HK2 (**S**), and cells expressing co-localisation of Panel 2 markers (**T**). Representative images of *n* = 3 at 10 × magnification shown

In placentas obtained from patients with preeclampsia (Fig. 2) or fetal growth restriction (Fig. 3) (*n* = 3 each group), mIF positivity for co-localisation of the trophoblast side-population markers was also performed. In both sets, as similarly observed to control placentas (Fig. 1), the panels of trophoblast side-population markers were only localised to select trophoblasts that bordered the basement membrane of the placental villi. There were no statistically significant changes to the cell count of cells that were positive for all assayed side-population markers (Supplementary Table I).

### Single-cell RNA Sequencing (scRNA-seq) in an Organoid Model of Trophoblast Differentiation

**Fig. 4:**
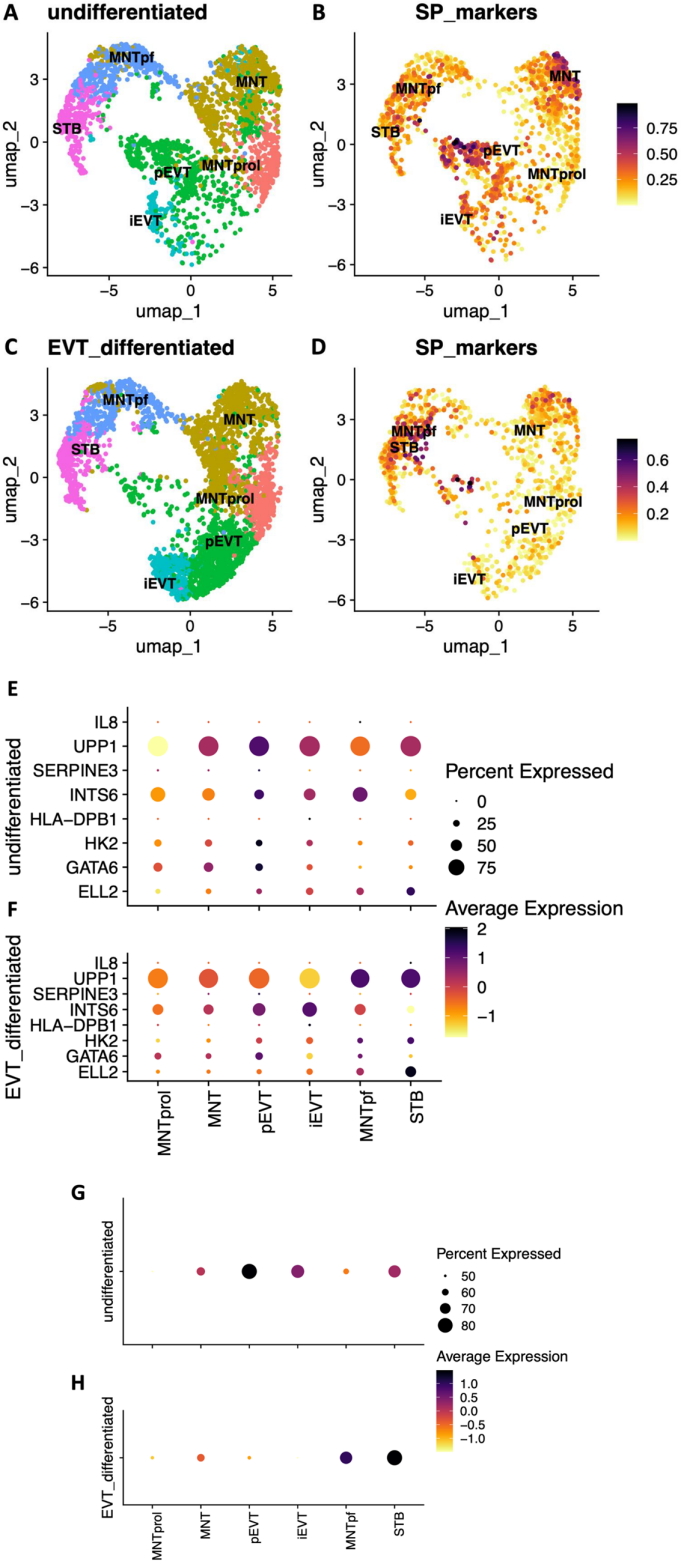
Single-cell RNA sequencing analysis of the trophoblast side-population-enriched genes in human trophoblast stem cell (hTSC) organoids and differentiated extravillous trophoblasts (EVTs). Transcriptomic analysis of a publicly available single-cell RNA sequencing dataset of *n* = 3 biological replicates of three-dimensional hTSC-derived organoids treated under hTSC conditions or induced to differentiate to EVTs for 21 days (Shannon et al., *Development* 2022). Analysis resulted in 6 distinct transcriptomic cell identities: mononuclear trophoblast (MNT), proliferative mononuclear trophoblast (MNTprol), pre-fusion mononuclear trophoblast (MNTpf), progenitor EVT (pEVT), invasive EVT (iEVT) and syncytiotrophoblast (STB). UMAP plot of cell identities in undifferentiated hTSC organoids (**A**). Feature plot showing a module score analysis for trophoblast side-population genes *CXCL8 (IL8), ELL2, GATA6, HK2, HLA-DPB1, INTS6, SERPINE3,* and *UPP1* where the relative expression of these genes is plotted (**B**)*.* UMAP plot of cell identities in differentiated EVT organoid culture (**C**). A feature plot showing a module score analysis for trophoblast side-population genes in EVT organoid culture (**D**). Dot plot of each trophoblast-side population marker gene expression to specific cell identities in undifferentiated (**E**) and differentiated EVT (**F**) organoid culture. Dot plot of the module scores for all trophoblast side-population panel markers in undifferentiated (**G**) and differentiated (**H**) organoid culture

To gain further insight in localising the side-population panel to point/s in trophoblast differentiation, expression of each gene was assessed in a publicly available single-cell RNA sequencing (scRNA-seq) dataset [15]. This dataset originated from *n* = 3 three-dimensional hTSC-derived organoids that were treated under hTSC conditions or induced to differentiate into EVTs. Processing of the single-cell RNA-sequencing data set resulted in 6354 cells that passed initial quality control. Cells were clustered in a uniform manifold approximation and projection (UMAP) plot according to transcriptionally similar states [36]. Transcriptionally similar cells and clusters were observed to be graphically closer in distance for undifferentiated (Fig. 4A) and EVT media-conditioned cultures (Fig. 4C). Each cluster was annotated with one of the following 6 cell identities: mononuclear trophoblast (MNT), proliferative mononuclear trophoblast (pMNT), pre-fusion mononuclear trophoblast (MNTpf), progenitor EVT (pEVT), invasive EVT (iEVT) and syncytiotrophoblasts (STB).

As a full panel, the 8 trophoblast side-population-enriched genes localised to most cells within undifferentiated organoid culture, (Fig. 4A-B, G) with dense expression mapping to cell identities including mononuclear trophoblasts, progenitor EVTs, invasive EVTs, and syncytiotrophoblasts. In contrast, in EVT differentiation conditions (Fig. 4C-D, H), these genes were enriched in a subset of cells undergoing syncytiotrophoblast differentiation (pre-fusion mononuclear trophoblasts and syncytiotrophoblasts), and in mononuclear trophoblasts.

Analysis localised each side-population gene to defined cell identities. *CXCL8 (IL8)* expression was restricted to select pre-fusion mononuclear trophoblasts in undifferentiated, and syncytiotrophoblasts in differentiated cell culture (Fig. 4E-F). *ELL2* was predominantly expressed in cell fusion-related identities (syncytiotrophoblast, pre-fusion mononuclear trophoblasts) under both conditions. Additional expression was observed in progenitor EVTs in undifferentiated hTSC-derived organoids. *GATA6* was expressed in subsets of progenitor EVTs, mononuclear trophoblasts and proliferative mononuclear trophoblasts across culture conditions*. GATA6* was also enriched in some pre-fusion mononuclear trophoblasts. *HK2* was expressed in progenitor EVTs under both states. Further expression in undifferentiated organoid culture was limited to select invasive EVTs, while expression was also observed in syncytiotrophoblast trajectories under EVT culture conditions (pre-fusion mononuclear trophoblasts and syncytiotrophoblasts). *HLA-DPB1* was expressed only in low numbers of invasive EVTs in both culture conditions. *INTS6* was surprisingly enriched in both culture conditions in cells associated with EVT differentiation (progenitor EVTs, invasive EVTs). *INTS6* also mapped to a subset of pre-fusion mononuclear trophoblasts in undifferentiated, and mononuclear trophoblasts in differentiated culture conditions. *SERPINE3* had enrichment in select progenitor EVTs and mononuclear trophoblasts, with additional expression in select undifferentiated proliferative mononuclear trophoblasts and differentiated syncytiotrophoblasts. *UPP1* expression was abundant in mononuclear trophoblasts and syncytiotrophoblasts across both groups, in undifferentiated EVT-related cell identities (progenitor EVTs and invasive EVTs), and finally in differentiated cell identity pre-fusion syncytiotrophoblasts.

### Trophoblast Side-population Gene Expression in Human Trophoblast Stem Cell (hTSC) Differentiation to Extravillous Trophoblast and Syncytiotrophoblast Phenotypes

To validate findings in scRNA-seq, we measured side-population marker mRNA expression in an *in vitro* model of trophoblast differentiation using hTSCs [30]. The timepoints examined were 0, 48 and 96 h post-differentiation in to the two main trophoblast lineages: extravillous trophoblasts (EVTs) and syncytiotrophoblasts.

**Fig. 5:**
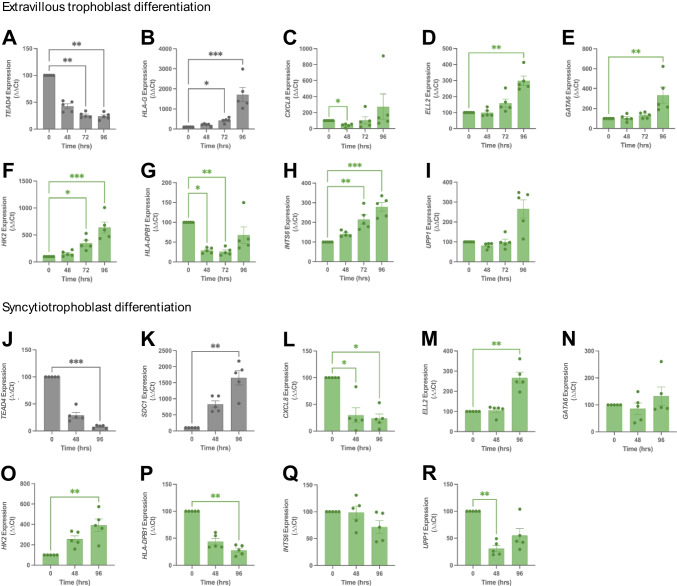
Trophoblast side-population-enriched genes and their expression with differentiation of human trophoblast stem cells (hTSCs) to extravillous trophoblasts and syncytiotrophoblasts. hTSCs were differentiated to extravillous trophoblasts (EVT) at 0, 48, 72 and 96 h post-differentiation, or syncytiotrophoblasts at 0, 48, 96 h post-differentiation. EVT differentiation was confirmed with *TEAD4* loss (*p* = 0.0067 72 h, *p* = 0.0018 96 h) (**A**) and induction of *HLA-G* expression (*p* = 0.026 72 h, *p* = 0.0002 96 h) (**B**). As hTSCs differentiated to EVTs, *CXCL8* expression was reduced (*p* = 0.030) (**C**). *ELL2* (*p* = 0.0047) (**D**), *GATA6* (*p* = 0.0039) (**E**) and *HK2* (*p* = 0.018 72 h, 0.0003 96 h) (**F**) were upregulated. *HLA-DPB1* expression reduced (*p* = 0.011 48 h, *p* = 0.0046 72 h) (**G**). *INTS6* was upregulated (*p* = 0.0092 72 h, *p* = 0.0005 96 h) (**H**). *UPP1* was unaltered (**I**). *SERPINE3* was not expressed at any timepoint (data not shown). Syncytiotrophoblast differentiation was confirmed with *TEAD4* loss (*p* = 0.0006) (**J**) and *SDC1* gain (*p* = 0.0011) (**K**). As hTSCs differentiated to syncytiotrophoblasts, *CXCL8* was reduced (*p* = 0.015 48 h, *p* = 0.012 96 h) (**L**). *ELL2* was upregulated (*p* = 0.0024 96 h) (**M**), *GATA6* was unaltered (**N**), *HK2* was raised (*p* = 0.0019) (**O**), *HLA-DPB1* was reduced at 48 h (with low statistical significance *p* = 0.051) and 96 h (*p* = 0.0031) (**P**). *INTS6* was unaltered (**Q**). *UPP1* expression was downregulated (*p* = 0.0063) (**R**). mRNA expression was normalised to the geometric mean of housekeeper genes. Data expressed as mean ± SEM with all experiments repeated *n* = 5 in triplicates for EVTs and duplicates for syncytiotrophoblasts. *p < 0.05, **p < 0.01, ***p < 0.001

Successful EVT differentiation was confirmed morphologically and via loss of the hTSC marker, *TEAD4* (Fig. 5A, *p* = 0.0067 at 72 h*,* *p* = 0.0018 at 96 h) and increases to EVT marker *HLA-G* (Fig. 5B, *p* = 0.026 at 72 h, *p* = 0.0002 at 96 h). *CXCL8* reduced briefly with EVT differentiation (Fig. 5C, *p* = 0.030 at 48 h post-differentiation). Meanwhile, *ELL2* (Fig. 5D, *p* = 0.0047 at 96 h), *GATA6* (Fig. 5E, *p* = 0.0039 96 h), and *HK2* (Fig. 5F, *p* = 0.018 at 72 h, *p* = 0.0003 at 96 h) increased with differentiation to EVTs. Conversely, *HLA-DPB1* expression was reduced (Fig. 5G, *p* = 0.011 at 48 h, *p* = 0.0046 at 72 h). *INTS6* was additionally upregulated with EVT differentiation (Fig. 5H, *p* = 0.0092 at 72 h, *p* = 0.0005 at 96 h). *UPP1* was unaltered with EVT differentiation (Fig. 5I), and *SERPINE3* expression was undetectable at all timepoints.

Syncytialisation was confirmed morphologically, and via loss of *TEAD4* expression (Fig. 5J, *p* = 0.0006 at 96 h) coupled with raised syncytiotrophoblast marker *SDC1* expression (Fig. 5K, *p* = 0.0011 at 96 h). While *CXCL8* reduced with differentiation (Fig. 5L, *p* = 0.015 at 48 h, *p* = 0.012 at 96 h), *ELL2* was upregulated (Fig. 5M, *p* = 0.0024 at 96 h), and *GATA6* was unaltered (Fig. 5N). *HK2* expression increased (Fig. 5O, *p* = 0.0019 at 96 h). *HLA-DPB1* was reduced at 48 h but did not hold statistical significance (Fig. 5P, *p* = 0.051) until 96 h post-differentiation (*p* = 0.0031). *INTS6* was unaltered (Fig. 5Q). Finally, *UPP1* was downregulated (Fig. 5R, *p* = 0.0063 at 48 h), while *SERPINE3* expression was also undetectable at all timepoints with syncytialisation.

### Trophoblast Side-population Gene Expression in Preeclampsia and Fetal Growth Restriction

**Fig. 6:**
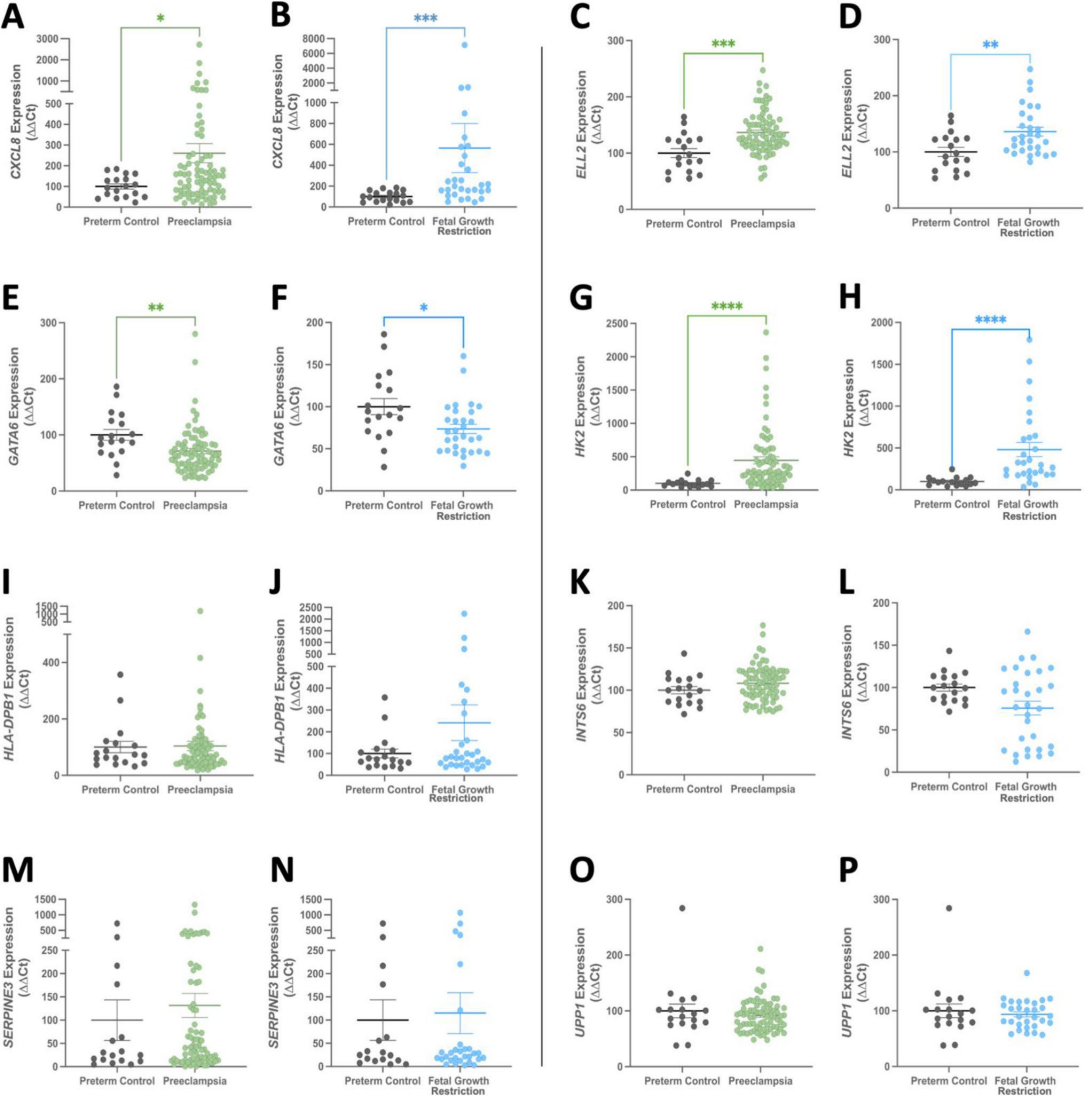
Trophoblast side-population-enriched genes *CXCL8*, *ELL2*, *GATA6*, and *HK2* are dysregulated in placentas obtained from pregnancies complicated by preeclampsia and fetal growth restriction. mRNA expression of side-population genes in placentas from participants with preeclampsia (*n* = 78 green circle), or fetal growth restriction (FGR, *n* = 30, blue circle) compared to gestation-matched preterm (< 34-week) controls (*n* = 18, grey circle). *CXCL8* was upregulated in preeclampsia (*p* = 0.037) (**A**) and FGR (*p* = 0.0001) (**B**). *ELL2* was elevated in preeclampsia (*p* = 0.0006) (**C**) and FGR (*p* = 0.0065) (**D**). *GATA6* was downregulated in preeclampsia (*p* = 0.0014) (**E**) and FGR (*p* = 0.0146) (**F**). *HK2* was upregulated in preeclampsia (*p* < 0.0001) (**G**) and FGR (*p* < 0.0001) (**H**). *HLA-DPB1* was unaltered in preeclampsia (**I**) and FGR (**J**); as was *INTS6* in preeclampsia (**K**) and FGR (**L**); *SERPINE3* in preeclampsia (**M**) and FGR (**N**); and *UPP1* in preeclampsia (**O**) and FGR (**P**). mRNA expression was normalised to the geometric mean of housekeeper genes. Individual symbols represent individual participants. Data is expressed as mean ± SEM. *p < 0.05, **p < 0.01, ***p < 0.001, ****p < 0.0001

Placental mRNA expression was examined for the 8 genes enriched in isolated trophoblast side-population cells (*CXLC8/IL8, ELL2, GATA6, HK2, HLA-DPB1, INTS6, SERPINE3, UPP1*) in placentas obtained from participants with early onset preeclampsia (*n* = 78) or fetal growth restriction (*n* = 30). Expression was compared to gestation-matched controls (*n* = 18). All genes were expressed in the placental samples. Of the 8 trophoblast side-population genes, *CXCL8* (Fig. 6A, *p* = 0.0335 in preeclampsia; Fig. 6B, *p* = 0.0001 in FGR) and *ELL2* (Fig. 6C, *p* = 0.0006; Fig. 6D, *p* = 0.0065 respectively) were upregulated. *GATA6* was downregulated in preeclampsia (Fig. 6E, *p* = 0.0014) and FGR (Fig. 6F, *p* = 0.0146). *HK2* was significantly upregulated in both conditions (Fig. 6G preeclampsia, Fig. 6H in FGR, *p* < 0.0001 both conditions). The remaining 4 genes were unaltered in both preeclamptic and FGR placentas (*HLA-DPB1,* Fig. 6I-J; *INTS6,* Fig. 6K-L; *SERPINE3,* Fig. 6M-N; and *UPP1*, Fig. 6O-P).

Placentas affected by concurrent preeclampsia and fetal growth restriction were included in each disease cohort. Thus, we also examined differences between those with one complication (only preeclampsia or only FGR) or both (concurrent preeclampsia and FGR) relative to controls (Supplementary Fig. 1). When stratified, *CXCL8* expression remained elevated in placentas obtained from FGR pregnancies, irrespective of concurrent preeclampsia (Supplementary Fig. 1A, *p* = 0.0003 FGR, *p* = 0.0033 FGR/preeclampsia). Conversely, no change was observed in those with preeclampsia alone. *ELL2* was unaltered in placentas from deliveries with FGR only. Its expression was elevated in the placenta from preeclamptic pregnancies, regardless of simultaneous FGR (Supplementary Fig. 1B, *p* = 0.0004 preeclampsia, *p* = 0.0023 FGR/preeclampsia). *GATA6* expression was reduced in placentas obtained from preeclamptic pregnancies, and in those that also delivered with FGR (Supplementary Fig. 1C, *p* = 0.0014 preeclampsia, *p* = 0.015 FGR/preeclampsia). Increased *HK2* was observed in all diseased groups but was most pronounced in placentas obtained from pregnancies complicated by preeclampsia (Supplementary Fig. 1D, FGR *p* = 0.015, FGR/preeclampsia *p* =  < 0.0001, preeclampsia *p* < 0.0001). While unaltered statistically in placentas from pregnancies complicated by FGR in initial analysis, when stratified, *INTS6* was markedly reduced (*p* < 0.0001), specifically in placentas affected by FGR alone. It remained unaltered in samples from FGR/preeclampsia and preeclampsia only (Supplementary Fig. 1F). *SERPINE3* and *UPP1* (Supplementary Fig. 1G, Supplementary Fig. 1H respectively) remained unaltered across each stratified disease cohort.

## Discussion

In this study, we sought to localise the 8 genes enriched in trophoblast side-population cells in the human placenta and characterise their expression in placental insufficiency. When analysed as individual genes, expression was induced following differentiation to EVTs or syncytiotrophoblasts. However, the power of the panel lies in the combined analysis. We visualised 6 of the 8 trophoblast side-population markers using multiplexed immunofluorescence, revealing localisation is likely restricted to rare trophoblast sub-populations, where contiguous villi may intersect. Analysis of single-cell sequencing data confirmed co-localisation of trophoblast side-population genes to a mononuclear trophoblast identity. Furthermore, of the 8 genes, 5 of them: *CXCL8, ELL2, GATA6* and *HK2,* and *INTS6* were found to be dysregulated in placenta obtained from pregnancies complicated by placental insufficiency.

We found that side-population markers were co-expressed in distinct trophoblast sub-types lining the basement membrane of the placental villi. These findings concur with current understanding of the residence of isolated human trophoblast stem cells [37]. In both disorders, dysregulated progenitor trophoblasts may contribute to homeostatic dysfunction, thereby initiating trajectories towards programmed cell death (apoptosis) [38]. Further insults to progenitor trophoblasts may impair differentiation processes. Downstream ramifications to effector functions may include villous formation and function [39]. Indeed, development of immature and aberrant placental villi that eventuate to a poorly perfused placenta and an impeded ability to perform as a specialised maternal-fetal interface has previously been identified [40, 41]. The current study identified a potential side-population of trophoblast cells localised to specific regions within the placental villi. Taken together with the transcriptomic analysis demonstrating enrichment in mononuclear trophoblast sub-types, this work may provide insight on the relevance of these cells and to how these processes may be disrupted in placental insufficiency. Future studies should importantly embark upon histological analysis beyond the villi that includes the anchoring columns where EVT differentiation occurs.

The side-population method results in a purified isolation of what are proposed to include trophoblasts higher in the lineage hierarchy, including trophoblast stem cells [42]. Isolation of side-population trophoblasts alongside cytotrophoblast isolation was recently used to generate organoids [43], providing a valuable tool to recapitulate the *in vivo* microenvironment of trophoblast stem cells more closely. Side-population cells are enriched in cancer stem cell populations and confer drug resistance to chemotherapeutics in other reproductive contexts including endometrial and breast cancer [44–46]. Investigating trophoblast side-population cells may therefore provide insights into their response to therapeutics, and interventions for placental insufficiency. Whilst the side-population method results in a more purified isolation of stem cells, it inevitably yields a heterogenous progenitor cell population [10]. This is reflected in the heterogenous cell type enrichment for side-population genes in our transcriptomic data. These findings emphasise that following studies must continue to interrogate the molecular governance of trophoblast stem cells that may be relevant to placental insufficiency pathogenesis. It would be intriguing to identify whether these 8 markers reflect the full spectrum or a unique subset [47] of a side-population trophoblast isolation, and then which subset, if any, contributes to placental insufficiency disorders.

While CXCL8 is a classical proinflammatory cytokine, it also has roles in stem cell activity in response to senescent microenvironments [48, 49]. Indeed, placental ageing is considered a pathogenic mechanism of placental insufficiency [2, 50, 51]. In the placenta, CXCL8 may be involved in trophoblast migration, proliferation, and invasion [52–54]. The elevated *CXCL8* observed in placentas obtained from FGR deliveries may indicate disruptions to these processes in a trophoblast side-population. *CXCL8* has previously shown to be raised in placentas of participants with established preeclampsia, with exacerbations in severe disease [55, 56]. Given minute expression in hTSC and EVT-related differentiation trajectories, and diffuse expression observed in immunofluorescent staining and hTSC *in vitro* differentiation studies, CXLC8 expression alone is likely not restricted to a specific placental cell type. It may therefore play multiple roles within the placenta.

*ELL2* encodes for a component of transcription elongation [57]. In our findings, *ELL2* expression was upregulated in placentas with established disease compared to their gestation-matched counterparts. Moreover, *ELL2* was downregulated in first trimester placentas obtained from chorionic villous sampling preceding a preeclampsia diagnosis [58]. The placenta-specific functions of ELL2 remain poorly understood [59]. It may inhibit wingless-related integration site (WNT) signalling, a key molecular pathway that directs progenitor function in the placenta and other processes in placental development [60]. In this study, ELL2 and GATA6 were unable to be localised as the Opal multiplexed immunofluorescence method was restricted to a maximum of 3 markers co-localised to any one cell in addition to DAPI nuclear counterstaining, with each representing a different subcellular compartment. Furthermore, the commercial antibodies available for these two markers displayed limited antibody specificity in the optimisation process.

The reduction in *GATA6* expression that appeared specific to preeclampsia provides a novel finding, as it has yet to be explored in the placenta or in pregnancy. In the intestine, where GATA6 is better characterised, GATA6 activates intestinal caudal type homeobox 2 (CDX2) expression. Here, it directs commitment to differentiation to the intestinal epithelial cell types and is dysregulated in gastrointestinal adenocarcinomas [61, 62]. In the placenta, CDX2 is involved in trophectoderm fate specification, and is instrumental to the maintenance of trophoblast stem cell populations [30, 63]. GATA6 may therefore have analogous placental roles to its intestinal function. Observed reductions to *GATA6* expression in preeclampsia and enrichment in trophoblasts committed to an EVT cell fate may therefore indicate aberrations in trophoblast specification.

This study demonstrated that placental *HK2* is elevated in both FGR and preeclampsia, with elevated expression in trophoblasts that have likely undergone a cell fate commitment. HK2 is a crucial glycolysis enzyme, and tightly regulates its balance with cell autophagy [64]. Intriguingly, HK2 activity itself is inhibited in a hypoxic environment. It may be important in the altered oxygen tension states in early pregnancy [65]. By extension, it may reflect compensatory mechanisms in response to the hypoxia and cell turnover observed in placental insufficiency disorders such as preeclampsia [66]. There have been confounding results in the field as to the direction of altered *HK2* expression in preeclampsia, with most findings observed in decidual tissue [60, 67–69]. HK2 activity is heightened with metabolic stress and maintains cancer stem cell potency [70]. The link between HK2 and trophoblast stem cell potency or response to environmental stress may be an interesting point for further research.

Until now, *INTS6* expression has not yet been explored in the placenta. Our findings show that *INTS6* expression is reduced exclusively in FGR. While FGR and preeclampsia share many pathophysiological aspects, the molecular governance of these processes has points of divergence. *INTS6* is more enriched in cells that have differentiated to terminal trophoblast phenotypes. INTS6 is known to inhibit proliferation via the WNT, protein kinase B (AKT) and extracellular signal-related kinase (ERK) signalling pathways [71–73]. Given that these pathways are also active in the placenta, INTS6 may function similarly here too. Reduced *INTS6* expression may therefore contribute to reduced cytotrophoblast proliferation that may occur in FGR [74].

Further steps beyond this study could be to isolate trophoblasts based on enrichment of trophoblast side-population markers, and to test whether they function similarly to the original trophoblast side-population isolated through the Hoechst method. Centrifugation speed, which may aid in purified isolation of potentially small-sized resident trophoblast stem cells, should be considered in these studies [75, 76]. Validating transcriptomic data with *in vitro* and *in vivo* experimentation would also be key to interpreting and furthering our understanding of mechanisms critical to trophoblast function, in both placental development and disease.

## Conclusion

We have limited understanding of each side-population marker in the placenta, and in placental insufficiency. This study identified the potential location of a unique trophoblast subset that is enriched for side-population markers. Furthermore, while each side-population marker alone may not be sufficient to determine progenitor function, it is important that all markers are considered collectively as a panel in future in depth functional studies. These findings contribute to the hypothesis that the trophoblast subtype indicated by side-population markers may represent a specific trophoblast identity that functions differentially to other trophoblast populations, and when dysregulated gives rise to placental insufficiency that leads to disorders of pregnancy including preeclampsia and/or FGR.

## Supplementary Information

Below is the link to the electronic supplementary material.

Supplementary file1 (PDF 1058 KB)

## Data Availability

All data from this study are available within the publication and supplementary files. Other resources used in this study are available from the corresponding author upon reasonable request. The full code used for single-cell transcriptomics is available upon request.
