# Supplementary material for: Trophoblast Side-Population Markers are Dysregulated in Preeclampsia and Fetal Growth Restriction
Source: Stem Cell Rev Rep. 2024 Jul 19;20(7):1954–70. doi: 10.1007/s12015-024-10764-w (PMC11445292; doi:10.1007/s12015-024-10764-w)
Supplement: Supplementary file 1 — Supplementary file1 (PDF 1058 KB) [file 12015_2024_10764_MOESM1_ESM.pdf]

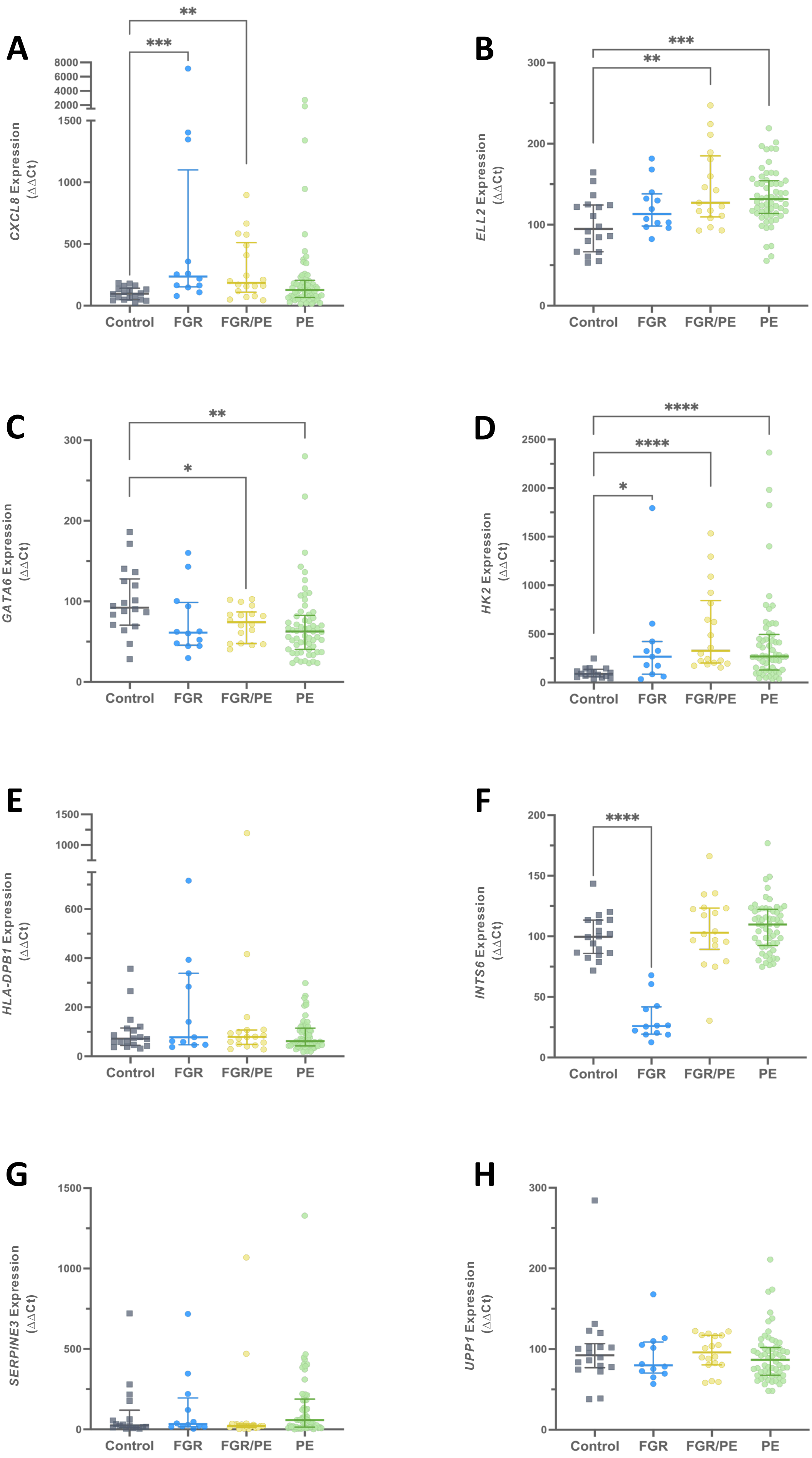

A
CXCL8 Expression
(ΔΔCt)
0
500
1000
1500
2000
4000
6000
8000
***
**
Control
FGR
FGR/PE
PE
B
ELL2 Expression
(ΔΔCt)
0
100
200
300
**
***
Control
FGR
FGR/PE
PE
C
GATA6 Expression
(ΔΔCt)
0
100
200
300
*
**
Control
FGR
FGR/PE
PE
D
HK2 Expression
(ΔΔCt)
0
500
1000
1500
2000
2500
*
****
****
Control
FGR
FGR/PE
PE
E
HLA-DPB1 Expression
(ΔΔCt)
0
200
400
600
1000
1500
Control
FGR
FGR/PE
PE
F
INTS6 Expression
(ΔΔCt)
0
50
100
150
200
****
Control
FGR
FGR/PE
PE
G
SERPINE3 Expression
(ΔΔCt)
0
500
1000
1500
Control
FGR
FGR/PE
PE
H
UPP1 Expression
(ΔΔCt)
0
100
200
300
Control
FGR
FGR/PE
PE

Supplementary Figure 1. Trophoblast side-population marker gene expression in placentas from single complication pregnancies (only preeclampsia or only fetal growth restriction) or pregnancies with concurrent preeclampsia/fetal growth restriction. mRNA expression of side-population-enriched genes in placentas from participants with only fetal growth restriction (FGR, n=13, blue circle), concurrent fetal growth restriction and preeclampsia (FGR/PE, n=17, yellow circle), or only preeclampsia (PE, n=61, green circle) compared to gestation-matched preterm (<34-week) controls (n=18, grey square). *CXCL8* was upregulated in FGR-only (p=0.0003) and concurrent cases (p=0.0033) **(A)**. *ELL2* was upregulated in concurrent cases (p=0.0023) and preeclampsia-only placentas (p=0.0004) **(B)**. *GATA6* was downregulated in concurrent (p=0.015) and preeclampsia-only placentas (p=0.0014) **(C)**. *HK2* was elevated in all conditions (p=0.015 FGR-only, p<0.0001 concurrent and preeclampsia-only) **(D)**. *HLA-DPB1* was unaltered under all conditions **(E)**. *INTS6* was reduced exclusively in placentas with only FGR (p<0.0001) **(F)**. *SERPINE3* **(G)** and *UPP1* **(H)** had no changes in mRNA expression between groups. mRNA expression was normalised to the geometric mean of housekeeper genes. Individual symbols represent individual participants. Data is expressed as mean ± SEM. *p<0.05, **p<0.01, ***p<0.001, ****p<0.0001.

Supplementary Table I. Whole slide multiplexed immunofluorescence (mIF) analysis for trophoblast side-population markers in human placental villous cross sections. Performed on n=3 biological replicates of preterm controls, preeclampsia and fetal growth restriction (2 panels of 3 markers each completed on serial section). Panel 1 consisted of HLA-DPB1, UPP1 and SERPINE3. Panel 2 consisted of CXCL8, INTS6, and HK2. Analysis conducted using HALO® Image Analysis Platform's Highplex FL module v4.1.3, with percentage (%) of total cells shown for each biological replicate, and mean ± SEM for each category.

**Category**	**Biological replicate**	**Panel 1 (% total cells)**				**Panel 2 (% total cells)**			
		**HLA-DPB1**	**UPP1**	**SERPINE3**	**Co-localisation**	**CXCL8**	**INTS6**	**HK2**	**Co-localisation**
**Preterm control**	1	6.44	57.42	50.98	1.06	59.53	96.66	97.22	57.90
	2	9.24	46.71	24.31	5.07	9.16	36.68	18.66	5.66
	3	0.78	44.42	44.11	0.28	16.77	90.01	93.99	15.44
Mean ± SEM		*5.49 ± 2.49*	*49.52 ± 4.01*	*39.80 ± 8.00*	*2.14 ± 1.48*	*28.49 ± 15.68*	*74.45 ± 18.98*	*69.96 ± 25.67*	*26.33 ± 16.03*
**Preeclampsia**	1	0.45	38.37	37.82	0.18	38.82	89.26	92.68	37.08
	2	4.29	61.32	51.29	3.35	36.06	46.24	61.87	22.28
	3	0.05	91.59	95.36	0.04	65.22	44.13	77.99	34.95
Mean ± SEM		*1.60 ± 1.35*	*63.76 ± 15.41*	*61.49 ± 17.38*	*1.19 ± 1.08*	*46.70 ± 9.29*	*59.88 ± 14.70*	*77.51 ± 8.90*	*31.4 ± 4.62*
**Fetal growth restriction**	1	5.19	67.00	40.14	3.57	84.28	86.65	76.64	67.57
	2	0.85	36.78	30.15	0.30	52.17	97.52	69.57	39.20
	3	1.14	68.72	68.55	0.59	85.13	93.60	96.57	78.75
Mean ± SEM		*2.39 ± 1.40*	*57.50 ± 10.37*	*46.28 ± 11.50*	*1.49 ± 1.05*	*73.86 ± 10.85*	*92.59 ± 3.18*	*80.93 ± 8.08*	*61.84 ± 11.77*

Supplementary Table II. Participant clinical characteristics <34-week gestation placental samples for preeclampsia, fetal growth restriction and gestation-matched controls. BMI = body mass index, SBP = systolic blood pressure and DBP = diastolic blood pressure. Mann-Whitney U tests used for comparison of medians. Chi-square tests used for categorical variables. BMI data missing for 4/18 control, 1/17 fetal growth restriction/preeclampsia (FGR/PE) and 8/61 PE samples. Smoking status data missing for 1/17 FGR/PE and 6/81 PE samples.

	Controls (n=18)	Fetal Growth Restriction (n=12)	Fetal Growth Restriction/ Preeclampsia (n=18)	Preeclampsia (n=61)	P-value
**Birthing Parent Age** (years)<br>Median (IQR)	32.5<br>(25.8 – 36.3)	30.5<br>(24.3 – 36.0)	31.0<br>(25.0 – 32.3)	31.0<br>(28.0 – 35.0)	$p_{FGR} = 0.569$<br>$p_{FGR/PE} = 0.246$<br>$p_{PE} = 0.865$
**Gestation at Delivery** (weeks)<br>Median (IQR)	30.4<br>(29.2 – 32.0)	31.4<br>(30.1 – 32.1)	29.9<br>(26.5 – 30.8)	30.0<br>(28.1 – 31.7)	$p_{FGR} = 0.225$<br>$p_{FGR/PE} = 0.175$<br>$p_{PE} = 0.505$
**BMI** (kg/m^2)<br>Median (IQR)	28.2<br>(24.8 – 35.1)	23.6<br>(19.3 – 30.5)	28.6<br>(25.6 – 35.5)	27.0<br>(24.0 – 36.1)	$p_{FGR} = 0.115$<br>$p_{FGR/PE} = 0.688$<br>$p_{PE} = 0.677$
**Parity** no. (%)<br>0<br>1<br>≥2	 4 (22.2)<br>9 (50.0)<br>5 (27.8)	 9 (75.0)<br>1 (8.3)<br>2 (16.7)	 15 (83.3)<br>2 (11.1)<br>1 (5.6)	 39 (63.9)<br>15 (24.6)<br>7 (11.5)	$p_{FGR} = 0.013$<br>$p_{FGR/PE} = 0.001$<br>$p_{PE} = 0.007$
**SBP at Delivery** (mmHg)<br>Median (IQR)	125.0<br>(117.3 – 130.0)	122.5<br>(116.3 – 132.3)	170.0<br>(155.0 – 180.0)	175.0<br>(165.0 – 181.5)	$p_{FGR} = 0.875$<br>$p_{FGR/PE} < 0.0001$<br>$p_{PE} < 0.0001$
**DBP at Delivery** (mmHg)<br>Median (IQR)	75.0<br>(70.0 – 80.0)	80.0<br>(71.3 – 84.5)	102.5<br>(93.8 – 110.0)	102.5<br>(100.0 – 110.0)	$p_{FGR} = 0.078$<br>$p_{FGR/PE} < 0.0001$<br>$p_{PE} < 0.0001$
**Birth weight** (g)<br>Median (IQR)	1587<br>(1298 – 2011)	993.5<br>(861.0 – 1110)	882.0<br>(581.0 – 1156)	1201<br>(887.0 – 1466)	$p_{FGR} = 0.0007$<br>$p_{FGR/PE} < 0.0001$<br>$p_{PE} = 0.0055$
**Assigned female at birth** no. (%)	8 (44.4)	5 (41.7)	11 (61.1)	28 (45.9)	$p_{FGR} = 0.880$<br>$p_{FGR/PE} = 0.317$<br>$p_{PE} = 0.913$
**Smoking status** no. (%)<br>Never<br>Quit<br>Current<br>Unanswered	 13 (72.2)<br>4 (22.2)<br>1 (5.6)<br>0 (0.0)	 6 (50.0)<br>4 (33.3)<br>2 (16.7)<br>0 (0.0)	 15 (83.2)<br>1 (5.6)<br>1 (5.6)<br>1 (5.6)	 50 (82.0)<br>2 (3.3)<br>3 (4.9)<br>6 (9.8)	$p_{FGR} = 0.410$<br>$p_{FGR/PE} = 0.384$<br>$p_{PE} = 0.044$
